# Steroids in chronic subdural hematomas (SUCRE trial): study protocol for a randomized controlled trial

**DOI:** 10.1186/s13063-017-1990-8

**Published:** 2017-06-05

**Authors:** Pierre-Louis Henaux, Pierre-Jean Le Reste, Bruno Laviolle, Xavier Morandi

**Affiliations:** 1 Department of Neurosurgery, Rennes University Hospital, 2, rue Henri Le Guilloux, 35033 Rennes, Cedex 9 France; 2 Department of Clinical Pharmacology, Rennes University Hospital, 2, rue Henri Le Guilloux, 35033 Rennes, Cedex 9 France; 3 Inserm, CIC 1414 Clinical Investigation Centre, Rennes University Hospital, 2, rue Henri Le Guilloux, 35033 Rennes, Cedex 9 France; 4 Rennes 1 University, Faculty of Medicine, 2 Avenue du Professeur Léon Bernard, 35043 Rennes, Cedex 4 France

**Keywords:** Chronic subdural hematoma, Corticosteroids, Methylprednisolone, Elderly patients, Conservative treatment

## Abstract

**Background:**

Chronic subdural hematoma (CSDH) is a common neurological pathology, especially in older patients. The actual “gold standard” of treatment is surgical evacuation, with various techniques used across neurosurgical teams. Over the years, there has been growing evidence that inflammatory processes play a major role in the pathogenesis of CSDH. In that context, the use of corticosteroids has been proposed alone or as an adjuvant treatment to surgery. However, this practice remains very empirical and there is a need for high-quality-of-evidence studies to clarify the role of corticosteroids in the management of CSDH.

**Methods/design:**

We propose a double-blind, randomized controlled trial comparing methylprednisolone versus placebo in the treatment of CSDH without clinical and/or radiological signs of severity. The treatment will be administered daily for a duration of 3 weeks, at a dose of 1 mg/kg. The primary endpoint will be the delay of occurrence of surgical treatment at 1 month following the introduction of the treatment. Secondary endpoints will include the rate of recourse to surgery, survival rate, quality of life and functional assessments, occurrence of systemic secondary effects and radiological assessment of the response to treatment. This multimodal assessment will be done at 1, 3 and 6 months. Two hundred and two patients (101 per arm) are expected to be included considering our primary hypotheses.

**Discussion:**

This trial started in June 2016; its results may open interesting alternatives to surgery in the management of patients harboring a CSDH, and may provide insights into the natural history of this common pathology.

**Trial registration:**

ClinicalTrials.gov, ID: NCT02650609. Registered on 4 January 2016.

**Graphical Abstract:**

Graphical output of the OBF boundaries.
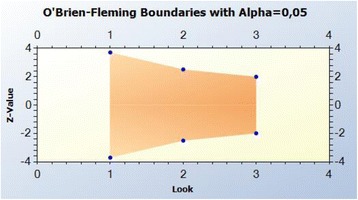

**Electronic supplementary material:**

The online version of this article (doi:10.1186/s13063-017-1990-8) contains supplementary material, which is available to authorized users.

## Background

Chronic subdural hematoma (CSDH) is one of the most frequent neurosurgical pathologies [1, 2]. To date, it has mainly involved older patients, with an annual incidence rate of 15 cases for 100,000 persons aged over 70 years [3, 4]. Considering that the population of patients aged over 65 years old is estimated to increase three-fold by the 2030s [5], and the increasing prescription of anticoagulant or platelet aggregation-inhibiting drugs, more and more practitioners will be confronted with this pathology which thus represents a major public health issue. Surgical evacuation of CSDH is currently the “gold standard” treatment [6]. In an often fragile population, surgical treatment carries significant morbidity at surgical (early recurrence which manifests itself in acute subdural hematoma, infections, intracerebral hematoma) as well as at medical (seizures, acute respiratory disease, urinary tract infections, confusional states, bedsore complications) levels. There is an increased mortality risk until 1 year after surgical evacuation [7], and hospital costs are high, with a very variable length of stay.

Beyond its classic traumatic etiology, the pathophysiology of CSDH involves inflammatory processes so that CSDH can now also be considered as an inflammatory disease. CSDH, therefore, appears to be a self-sustaining process of neoangiogenesis and fibrinolysis, leading to progressive increase in hematoma volume [8–19]. In this context, corticosteroids could represent an alternative treatment for CSDH because of their inhibiting effects on vascular endothelial growth factor, tissue plasminogen activator, interleukin-6, interleukin-8 and platelet-activating factor [20]. However, the scientific literature on the subject is still very limited [21]. In fact, only case reports and preliminary nonrandomized or retrospective studies have suggested the efficacy of steroids alone or as adjuvant therapy to surgery [22–26].

In this context, a double-blind randomized study is mandatory to assess the efficacy of corticosteroids in CSDH. We propose a randomized controlled trial aiming at evaluating the efficacy of corticosteroid treatment in patients with CSDH without clinical or radiological signs of severity. The investigational medicinal product is methylprednisolone, a synthetic glucocorticoid widely used in treating various inflammatory diseases. It is a commonly used drug for which the mechanisms of action, drug interactions and adverse effects are well known. The purpose of this article is to describe the rationale for the study and to reveal the protocol of the SUCRE (Steroids in sUbdural ChRonic hEmatomas) trial.

## Methods/design

### Study design

The proposed study is a prospective, multicenter, double-blind, randomized controlled, (methylprednisolone versus placebo) phase-III superiority trial performed on two parallel groups. The primary objective is to evaluate the efficacy of corticosteroid treatment in patients with CSDH without clinical or radiological signs of severity. Secondary objectives are to assess the effect of methylprednisolone on quality of life evolution, morbidity and mortality, and radiological evolution of the lesions. This trial is registered at ClinicalTrials.gov as NCT02650609. This article has been written following the Standard Protocol Items: Recommendations for Interventional Trials (SPIRIT) guidelines [27] (Additional file 1).

#### Primary endpoint

The primary evaluation criterion is the delay of occurrence of surgical treatment (censored criteria) of the CSDH at 1 month. One month has been chosen for assessing the primary endpoint because most surgical interventions, if needed, are expected to occur during the first month following the diagnosis of CSDH. Patients who are nonevaluable for the primary endpoint (death, loss to follow-up, etc.) will be censored at the date of death or the latest news.

Criteria for recourse to surgery are (one is sufficient): lack of clinical improvement at 1 month, immediate or delayed clinical deterioration, appearance of radiological signs of severity (midline shift >5 mm, uncal transtentorial herniation).

#### Secondary endpoints

Secondary evaluation criteria are: quality of life assessed by the Short Form 12-item (SF12) scale [28] at 1, 3 and 6 months; time to surgical treatment during the first 6 months (censored criteria); rate of surgical treatment of the CSDH at 1, 3 and 6 months; functional scales: daily living (Instrumental Activities of Daily Living; IADL) [29], cognitive (Mini-Mental State Examination; MMSE) [30] and the modified Rankin Scale [31] at 1, 3 and 6 months; plasma sodium, potassium and fasting glucose at days 0, 7, 14 and 21 and at 1 month; occurrence of adverse events potentially related to methylprednisolone (electrolyte disorders: hypokalemia, metabolic alkalosis, sodium retention (edema), hypertension, congestive heart failure; endocrine and metabolic disorders: Cushing’s syndrome, adrenal insufficiency, glucose disorders (impaired glucose tolerance, diabetes); gastrointestinal disorders: ulcer, perforation, gastrointestinal tract hemorrhage; skin disorders: acne vulgaris, purpura, hematoma, hypertrichosis; neuropsychological disorders: euphoria, insomnia, agitation, confusion, depression; ophthalmological disorders such as glaucoma); survival at 6 months; radiological improvement, defined by reduction of maximal thickness of hematoma and reduction of midline shift evaluated at 1, 3 and 6 months.

Subgroup analyses of all study endpoints will also be performed in subacute (less than 4 weeks old) and chronic (more than 4 weeks old) CSDH, and in patients treated or not treated with anticoagulant or platelet antiaggregant therapy.

### Description of the measures taken to reduce and prevent bias

#### Randomization

The randomization list will be prepared by the Biometrics Unit of the Clinical Investigation Centre Inserm 1414 of Rennes. Randomization will be stratified by center and equilibrated at a 1:1 ratio. After verification of selection criteria during the screening phase, the investigator will proceed to the randomization of the patient the day of baseline (D0). The investigator sends the order-processing request to the pharmacist of his center. The pharmacist attributes treatment to the patient according to their list in chronological order and informs the coordinating centre by faxing the completed order.

#### Methods of blinding

This study is double-blind. Active treatment and placebo will be identical and prepared in capsules by an institutional platform of pharmaceuticals production at Brest University Hospital.

### Study population

Our hypothesis is that 80% of the patients taking placebo will be operated on at 1 month. A total sample size of at least 202 patients (142 events) is required to achieve 90% power to detect a decrease in the rate of surgery to 60% (hazard ratio of 1.756) with methylprednisolone, using a two-sided log-rank test with a 0.05 significance level assuming that the hazards are proportional (nTerim, V1.1, Statistical Solutions Ltd., Cork, Ireland).

#### Inclusion criteria

Patients eligible for the study must follow the following criteria: age above 18 years old; with chronic or subacute, uni- or bilateral subdural hematomas; confirmed by cerebral computed tomography (CT) scan without contrast enhancement (an additional CT scan is mandatory for patients with CSDH diagnosed using another imaging modality (e.g., magnetic resonance imaging)); without clinical (Glasgow Coma Scale score ≤12, motor deficit <4/5) and radiological signs of severity (midline shift >5 mm, uncal transtentorial herniation) assessed by the neurosurgeon investigator in charge of the patient; written informed consent from patients or their next of kin according to the patients’ cognitive status.

#### Exclusion criteria

The noninclusion criteria of this trial (one criterion is enough to exclude a patient) are the following: clinical signs of severity (Glasgow Coma Scale score ≤12, motor deficit <4/5); radiological signs of severity (midline shift >5 mm, uncal transtentorial herniation); diabetes mellitus; contraindications for methylprednisolone (uncontrolled infectious disease, evolutive viral disease (e.g., HIV, hepatitis, herpes, varicella, herpes zoster), known psychiatric disorder, known hypersensitivity to methylprednisolone or lactose intolerance); previous surgery for CSDH during the past 6 months; pre-existing severe dementia, defined by a MMSE score <16 related to an etiology other than CSDH; neurological pathology that can be associated with dementia; long-term corticosteroid treatment; patient under legal protection (conservatorship, trusteeship, guardianship) or deprivation of freedom; participation of the patient in other concomitant clinical research studies.

### Treatment administered in the study

#### Identification of treatment

Methylprednisolone will be supplied as a powder and encapsulated in capsules containing 16 mg of drug with lactose as the excipient. Placebo capsules contain only lactose. Capsules will be packaged in boxes of 40. Boxes will be stored in containers of three boxes.

#### Packaging and labeling

Each box of medication for the study is identified by a specific label for a clinical trial in compliance with applicable regulatory requirements including European regulations.

#### Administration

The duration of treatment is 21 days. Capsules are administered orally in the morning, during breakfast. They will be dispensed by a nurse when patients may not be able to take capsules themselves. Prescription dosage is adapted according to the weight of the patient (approximately 1 mg/kg): three 16-mg capsules/day (patient <60 kg), four 16-mg capsules/day (patient 60–80 kg), five 16-mg capsules/day (patient >80 kg).

The treatment duration is supported by the pathophysiology of CSDH formation (inflammatory process supposed to be maximal at 2 to 3 weeks) [4, 8, 9, 11–19].

#### Precautions for use

Because of the potential risk of water retention, hyperglycemia or hypokalemia, the patients will receive written dietetic advice to follow a diet with a low intake of fast-release carbohydrates and salt, and increased intake of potassium, during the treatment period. A specific dietetic form will be drawn up by a dietician before the beginning of the study. In addition to these dietetic measures, specific monitoring of plasma sodium, potassium and glucose will be carried out. Patients will also be informed of all expected steroid side effects and will be advised to contact the investigator in case of suspicion of any adverse effects during the treatment period.

#### Authorized and unauthorized medicinal products

Management of drugs affecting hemostasis (antiplatelet drugs, orally administered anticoagulants and heparin) will be based on the habits of the participating centers in the absence of evidence-based guidelines. Contraindications to medicinal product combinations listed in the Summary of Product Characteristics for methylprednisolone, will be complied with. In addition, clinical and/or close laboratory test monitoring will be applied whenever a potential interaction with a concomitant medicinal product exists.

In case of the failure of treatment with corticosteroids, defined by a lack of clinical improvement, clinical deterioration, immediately or after a phase of improvement, radiological progression or corticosteroid intolerance, surgical treatment should be considered. In case of persistence of the CSDH at the end of the study, its treatment will follow the centre’s normal policy.

### Measures

#### Screening phase

The screening phase takes place during the hospitalization or consultation in a neurosurgical unit during which the diagnosis of CSDH is confirmed. A standard clinical examination is performed to ensure that the patient meets the selection criteria of the study. The initial CT scan (performed onsite or in another hospital depending on the origin of the patient) completes this initial patient screening. If the patient is eligible, written informed consent from the patient or a representative (if the patient’s cognitive state does not allow informed decision) is necessary before enrollment.

#### Baseline data collection

The following information is collected before initiation of the treatment:

Patients’ characteristics: sex, age, weight, height, major medical history (including chronic alcoholism), ongoing medications affecting hemostasis (anticoagulants and platelet antiaggregants), and symptoms that led to the diagnosis of CSDH (persistent headache, repeated falls, balance disorders, attention and memory disorders, muscular weakness, motor or sensitivity disorders).

The clinical assessment includes: blood pressure measurement, neurological examination (Glasgow Coma Scale (GCS) score, diameter and symmetry of the pupils, presence of a focal neurological deficit), functional scales (modified Rankin Scale, IADL and MMSE assessments, quality of life (SF12 scale) and comprehensive review of the overall clinical assessment score with the ASA (American Society of Anesthesiologists).

#### Follow-up

**Fig. 1:**
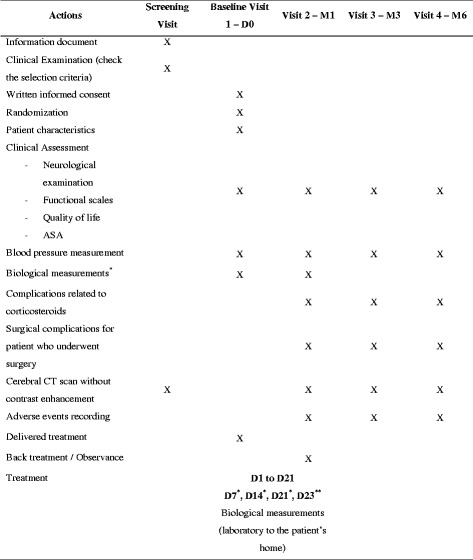
Study flow chart. *Plasma sodium, potassium, and fasting glycemia/**fasting plasma cortisol

Patients are followed up for a period of 6 months, and are evaluated at 1, 3 and 6 months. At days 0, 7, 14 and 21 and at the 1-month visit, plasma sodium, potassium and fasting glucose are measured. Fasting plasma cortisol at 8 a.m. is measured 2 days after the end of the treatment with corticosteroids (at day 23). Patients will undergo interval cerebral CT scans to evaluate their CSDH at the following intervals: screening visit, M1, M3 and M6 (Fig. 1). This biological surveillance allows the detection of any electrolyte or glycemic impairment that could occur with steroid treatment.

### Statistical analysis

Statistical analysis will be performed on all evaluated patients (intention-to-treat analysis in the methodology/biometrics unit of the Clinical Investigation Centre Inserm 1414 of Rennes. The statistical significance will be considered to be *p* values < 0.05 for all analysis.

#### Analysis of the primary endpoint

Time to surgical treatment (i.e., the delay of occurrence of surgical treatment) of the CSDH at 1 month will be compared between the two groups with the log-rank test. Two interim analyses after inclusion of one third and two thirds of the patients, and one final analysis are planned. Stopping rules will use the alpha spending function with the O’Brien-Fleming boundary. The cumulative values of alpha for each analysis are: 0.00021 at the first analysis, 0.01202 at the second analysis and 0.04626 at the final analysis (nTerim, V4.0, Statistical Solutions Ltd., Cork, Ireland). The trial will be stopped early if the significance of the log-rank test is below these alpha values.

#### Analysis of other criteria

Student’s *t* test or a Mann-Whitney test if necessary will be used to compare quantitative variables. Quantitative criteria evaluated several times during the monitoring will be compared with repeated measures analysis of variance (ANOVA) with two factors (time, group). For qualitative criteria, a chi^2^ test will be used. For all these analyses, adjustments can be made in case of heterogeneity at inclusion.

#### Analysis of adverse events

Possible adverse events are coded according to the MedDRA classification and are the subject of a descriptive analysis.

### Data Safety Monitoring Board

An independent Data and Safety Monitoring Board is constituted before study initiation and comprises five members who are not involved in the study, including a neurosurgeon, a neurologist, an endocrinologist, a pharmacologist and a methodologist. The Data and Safety Monitoring Board meets after each interim analysis and at the end of the study. It can also meet on the request of the coordinating investigator or the methodologist if serious adverse events, or results which can jeopardize the existence of the protocol, occur. The Data and Safety Monitoring Board can propose to stop the study if interim statistical analyses reach significance or if it appears that study continuation would be contrary to ethics rules for safety reasons, or if the publication of trial results yields data that proves our hypothesis.

## Discussion

Although corticosteroids have previously been used in CSDHs, this practice has never been evaluated with a well-designed, high-quality-of-evidence study. Indications, duration and dosage and type of corticosteroid greatly vary depending on the habits of each team. In that context our study will help to clarify the prescription procedure.

Under physiological conditions the subdural space is a virtual space with histological continuity between the dura and the arachnoid. Various conditions, especially head injury, can lead to the separation of these two membranes. A proliferation of parietal cells can then occur in the subdural space, resulting in the formation of fibrous membranes in which capillaries proliferate under the influence of vascular endothelial growth factor [32]. These capillaries have inflammatory and permeable walls, and are partially responsible for the production of the subdural fluid [33]. This leakage contains partially degraded erythrocytes and coagulation factors, but the subdural liquid is uncoagulable because of the presence of large quantities of tissue plasminogen activator [34], a fibrinolytic enzyme. Inflammatory mediators, such as interleukin-6 and interleukin-8, platelet-activating factor and bradykinin [9] are also present in the subdural fluid, contributing to the persistence of inflammation.

With regard to these pathophysiological aspects of CSDH, corticosteroid use has been proposed to treat CSDH. The existing studies used varied methodologies, and had a low quality of evidence as demonstrated by a recent systematic review [35]. In a small study comparing patients treated with surgery (82 cases) and dexamethasone (26 cases), only one patient in the group with dexamethasone required secondary surgery [25]. In a retrospective series of 122 patients, only 20% of patients receiving dexamethasone prior to surgery required secondary surgery and the medical complications of treatment were essentially mild hyperglycemia [23]. In another retrospective study including 496 patients, preoperative corticosteroid administration seemed to reduce the recurrence rate of CSDH after burr-hole craniostomy without increasing the incidence of complications and treatment-related deaths [21]. A retrospective study testing the efficacy of corticosteroids as adjuvant therapy to surgery (142 patients with adjuvant corticosteroids and 56 patients without adjuvant corticosteroids) showed a trend toward a survival benefit with medical treatment (risk of death three-fold less in the case of adjuvant corticosteroids (*p* = 0.006)) [24]. Despite this data, there is a lack of published evidence concerning the efficacy of corticosteroids in reducing the rate of surgery. We have selected the 60% reduced rate of surgery based on our empirical experience with this treatment for this pathology.

Corticosteroids have the advantage of being well known and widely used for various pathologies. We arbitrarily chose to use methylprednisolone rather than dexamethasone because in France it is the most commonly used corticosteroid in neurosurgical pathologies. Even if diabetes is not a strict contraindication for the administration of corticosteroids, we added the presence of pre-existing diabetes as an exclusion criterion. We considered that the risk of including glycemic disorders with potentially severe consequences would exceed the benefit of avoiding surgery.

As mentioned above, one of the weaknesses of our work is the lack in the academic literature of studies describing the natural history of CSDH without treatment; some may claim that some cases of CSDH without clinical and neurological signs of severity could heal spontaneously. Another issue concerns the inclusion of patients with cognitive disorders. Confusion and dementia might be difficult to distinguish in an acute context, so the questioning of the relatives is of particular importance. We still wanted to include patients with doubtful or mixed semiology, who pose the greatest ethical dilemmas in everyday practice. A medical alternative to surgery in these cases would be of particular interest considering the invasiveness of surgery for that fragile population. However, corticosteroids might have psychic side effects liable to destabilize the mental status of such patients.

Another point of debate is the duration of corticosteroid therapy. After discussion with the endocrinology team of our establishment and on the basis of the academic literature [36–39], we decided to limit the treatment to 3 weeks. Indeed, after this period the risk of adrenocortical insufficiency induced after the discontinuation of corticosteroids increases significantly. Usually, adverse effects associated with corticosteroids predominantly occur during consumption of these medications over several weeks or months. We also decided to lower the inclusion boundary to a GCS score of 13. For those patients with a GCS score of 13 and, therefore, mild vigilance disorders, we recommend close monitoring in neurosurgical departments during the first days or the first week of treatment in order to assess the effectiveness of the treatment and possibly perform surgery in case of clinical deterioration. We left the choice to each of the investigators about the choice of timing to perform a secondary surgery.

Surgical drainage of CSDH finally remains the gold standard treatment. Even if the surgical procedure is simple, it can carry significant morbidity and mortality [7], especially in older patients with significant medical history. A medical alternative to surgery seems particularly relevant in these patients. Several surgical techniques are described in the academic literature. All these techniques aim at relieving the mass effect on the brain and try to minimize the risk of recurrence. The evacuation may be performed by craniotomy or by a single burr hole. Others propose a craniostomy allowing an evacuation of the hematoma in “closed skull” conditions [40]. The common goal of every technique is to wash maximally the subdural space while minimizing pneumocephalus. To perform this, the only technique that has been validated with a good level of evidence is the drainage of the subdural space, showing a recurrence rate decreasing from 24 to 9% with a subdural drain [41]. We left the choice of the surgical technique to each participating center, as well as the type of anesthesia and additional post-operative treatments (for example, the use of anticonvulsants). The perioperative management of anticoagulant and antiplatelet drugs is a particularly complex matter in the management of CDSH patients. Although these medications might be given for vital reasons, surgical treatment often requires their temporary suspension or decrease which could expose patients to cardiological or thromboembolic events. Our protocol leaves the choice to investigators to continue or discontinue these medications based on their team habits. The ideal management would be to treat patients with corticosteroids without stopping their antiplatelet or anticoagulant therapies. A specific study with high standard of proof would be useful to determine a clearer strategy on the management of these treatments in CDSH patients. In the meantime, subgroups analysis of this study might give some insights on that particular point.

## Trial status

This study protocol concerns an ongoing trial that has not completed patient recruitment at the time of submission.

## Additional file

Additional file 1: SPIRIT Checklist for the SUCRE trial. (DOC 129 kb)

## Abbreviations

IADL: Instrumental Activities of Daily Living
MMSE: Mini-Mental State Examination
